# Oncologist use of the Adjuvant! model for risk communication: a pilot study examining patient knowledge of 10-year prognosis

**DOI:** 10.1186/1471-2407-9-127

**Published:** 2009-04-28

**Authors:** Jeffrey K Belkora, Hope S Rugo, Dan H Moore, David W Hutton, Daniel F Chen, Laura J Esserman

**Affiliations:** 1 Department of Surgery, University of California San Francisco, San Francisco, California, USA; 2 Department of Medicine, University of California San Francisco, San Francisco, California, USA; 3 Department of Epidemiology and Biostatistics, University of California San Francisco, San Francisco, California, USA; 4 Department of Management Science and Engineering, Stanford University, Stanford, California, USA

## Abstract

**Background:**

Our purpose was to collect preliminary data on newly diagnosed breast cancer patient knowledge of prognosis before and after oncology visits. Many oncologists use a validated prognostic software model, Adjuvant!, to estimate 10-year recurrence and mortality outcomes for breast cancer local and adjuvant therapy. Some oncologists are printing Adjuvant! screens to use as visual aids during consultations. No study has reported how such use of Adjuvant! printouts affects patient knowledge of prognosis. We hypothesized that Adjuvant! printouts would be associated with significant changes in the proportion of patients with accurate understanding of local therapy prognosis.

**Methods:**

We recruited a convenience sample of 20 patients seen by 2 senior oncologists using Adjuvant! printouts of recurrence and mortality screens in our academic medical center. We asked patients for their estimates of local therapy recurrence and mortality risks and counted the number of patients whose estimates were within ± 5% of Adjuvant! before and after the oncology visit, testing whether pre/post changes were significant using McNemar's two-sided test at a significance level of 5%.

**Results:**

Two patients (10%) accurately estimated local therapy recurrence and mortality risks before the oncology visit, while seven out of twenty (35%) were accurate afterwards (p = 0.125).

**Conclusion:**

A majority of patients in our sample were inaccurate in estimating their local therapy recurrence and mortality risks, even after being shown printouts summarizing these risks during their oncology visits. Larger studies are needed to replicate or repudiate these preliminary findings, and test alternative methods of presenting risk estimates. Meanwhile, oncologists should be wary of relying exclusively on Adjuvant! printouts to communicate local therapy recurrence and mortality estimates to patients, as they may leave a majority of patients misinformed.

## Background

In 2007, an estimated 178,480 new cases of invasive breast cancer were diagnosed among US women and approximately 40,460 died from the disease [1]. To reduce the risks of progression, recurrence, and ultimately mortality, adjuvant therapies such as chemotherapy and hormone therapy are often recommended in addition to local therapies such as surgery and radiation.

These therapies have uncertain outcomes. For example, prior studies indicate that some chemotherapy regimens carry approximately a 0.5% chance of treatment-induced leukemia [2] in addition to increases in 10-year absolute survival ranging from 2–11% for most breast cancer patients [3]. Oncologists must communicate these and other uncertain outcomes to patients in order to assure that treatment decisions are made based on valid information and well-considered preferences.

Risk communication includes, among other elements, "information about the nature and likelihood" of key outcomes of interest [4]. In order to facilitate oncologist communication about the likelihoods of treatment outcomes, researchers have created prognostic models based on registry outcomes data and evidence from clinical trials. One such model, Adjuvant!, is used by 44% of community oncologists and 78% of clinical research oncologists "to estimate breast cancer patients' risk of recurrence and/or mortality [5]." Adjuvant! forecasts recurrence and mortality risks based on specific patient and tumor characteristics, and estimates the treatment benefit of various treatment options, ranging from local therapy only to combined chemotherapy and hormone therapy [6-8]. Adjuvant! has been validated against a Canadian database of outcomes [9].

Some oncologists have begun printing screenshots from the Adjuvant! risk estimation tool, and discussing the estimates with patients during oncology visits. One study has documented that this practice is associated with fewer early-stage patients choosing chemotherapy [10,11]. It is not known, however, whether Adjuvant! printouts achieve their intended purpose of informing breast cancer patients about the likelihood of 10-year recurrence and mortality outcomes. Therefore, we embarked on a pilot study to collect preliminary data about patient knowledge regarding local therapy recurrence and mortality risks, and whether patient knowledge might be influenced by oncologist use of Adjuvant! printouts during the oncology visit. We focused in this study on local therapy prognosis, i.e. Adjuvant!'s 10-year recurrence and survival estimates, because these estimates represent a baseline that patients should understand in order to make informed decisions about whether to take systemic adjuvant therapy.

We hypothesized that Adjuvant! printouts in the context of an oncology visit should be associated with significant changes in the proportion of patients with accurate understanding of local therapy prognosis. We were open to the possibility that patients might be more confused than enlightened by Adjuvant!'s screens, which were originally designed as a reference tool for oncologists.

We asked two questions about patient estimates of local therapy recurrence and mortality rates:

1. Did patient estimates of local therapy recurrence and mortality risks match Adjuvant! estimates before the oncology visit?

2. Did patient estimates of local therapy recurrence and mortality risks move closer to Adjuvant! after the oncology visit?

Our study is the first to report on patient knowledge of local therapy recurrence and mortality risks in the context of using Adjuvant!

## Methods

### Study design and settings

The study design was a single-arm, exploratory, hypothesis-generating pilot study of a convenience sample of 20 consecutive patients consulting two senior medical oncologists at the University of California, San Francisco (UCSF) Breast Care Center after having seen one surgeon. The study was approved by the UCSF Committee on Human Research and the Department of Defense Human Subjects Research Review Board, and informed consent was obtained from each participant. The consent form explained that patients would receive printed, quantitative information about their prognosis under different treatment scenarios, and alerted them that they would be asked to complete a short questionnaire assessing their understanding of the materials. Patients were not primed as to the specifics of the measurements or instruments.

**Figure 1:**
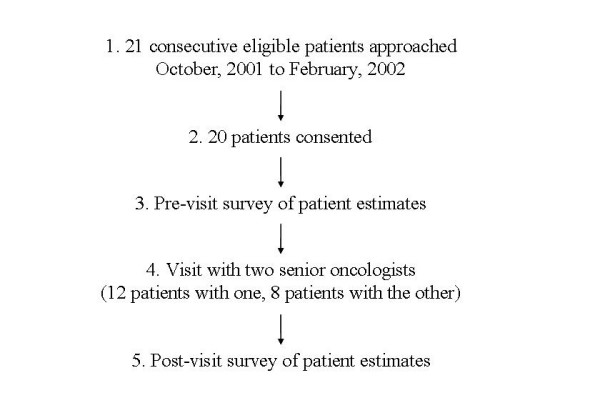
**Study schema**. Study schema showing chronological steps in this pre/post single-arm study.

The two participating oncologists were involved in the general design of the study, including the selection of the primary outcome and measure of patient knowledge. Prior to the study, the oncologists caucused to standardize their use of the intervention. They agreed to see all study patients in 60 minute visits. Half of each visit was devoted to history taking, a physical exam, and disclosure of treatment risks and side effects. The oncologists presented consistent information about treatment risks and side effects based on systematic reviews. The second half of the visit was devoted to reviewing Adjuvant! printouts showing recurrence and mortality rates as a function of the patient's situation, and answering patient questions about the printouts. Patients were eligible to participate in the study if they could speak and read English, if they had completed surgery for stage I, II, or IIIa breast cancer, if they had not initiated any form of adjuvant therapy, and if their medical charts included tumor size, tumor grade, hormone receptor status, node status, and age. Patients were not eligible to participate in the study if they had metastatic disease, if they needed further surgery to complete staging, or if they were unable to provide informed consent. Patients were enrolled between October, 2001 and February, 2002. (Figure 1)

### Intervention and procedures – Adjuvant! printouts

**Figure 2:**
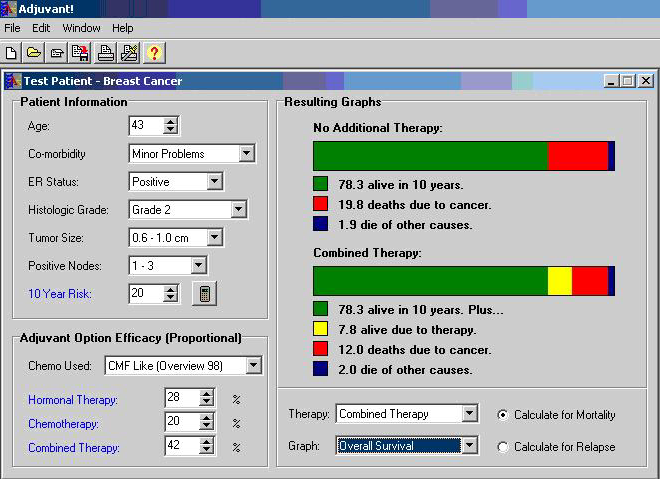
**Screenshot of the Adjuvant! software program**. The two oncologists in this study used Adjuvant! to generate prognostic estimates tailored to patient information shown in the upper left of the screen. Oncologists printed screenshots showing patient recurrence (relapse) or mortality, selected at bottom right, for "no additional therapy", top right.

The intervention in this single-arm, pre/post study consisted of a printout of the graphs from the Adjuvant! software program, which presented estimates of the patient prognosis based on patient-specific inputs consisting of age, tumor size and grade, estrogen receptor status, node status, and number of comorbidities. (See Figure 2). A study coordinator generated the printout upon configuring the Adjuvant! inputs in discussion with the oncologist prior to the patient-oncologist consultation. During the patient-oncologist visit, each of the two oncologists used the printout as a visual aid to communicate mortality and recurrence information.

### Outcomes and instruments

Our primary outcome was patient knowledge of both recurrence and mortality risks because, although these outcomes are correlated, patients experience recurrence as being a threat to quality of life in addition to mortality being a threat to length of life. We focused on local therapy prognosis (prognosis after surgery with or without radiation) because this represents a baseline from which patients and doctors should weigh the further benefits and risks of adjuvant chemotherapy or hormone therapy.

We focused on an accuracy standard of ± 5% absolute percentage points because Adjuvant! was found in a validation study to be accurate within 2–5% in estimating local and adjuvant therapy mortality and recurrence risks for a broad range of breast cancer cases [9]. In addition, 10-year absolute mortality benefits of chemotherapy range from 2 to 11% for a wide range of breast cancer cases [3]. Therefore we selected ± 5% as a clinically significant threshold representing the maximum allowable margin of error.

The instrument for measuring patient beliefs regarding 10-year recurrence and mortality risks was a survey administered before and after the patient-oncologist consultation. The items included:

The chance of my breast cancer returning or spreading within the next 10 years after having local therapy is...

The chance that I will die from my breast cancer within the next 10 years after having local therapy is...

The response format was a list of 21 potential responses (0%, 5%, 10%, ... to 100% in increments of 5%).

### Analyses

1. *Did patient estimates of local therapy recurrence and mortality match Adjuvant! estimates before the oncology visit?*

To answer this question, we calculated the difference between Adjuvant! and patient estimates and assessed the number of patient estimates that were within ± 5% of their Adjuvant! estimate.

2. *Did patient estimates of local therapy recurrence and mortality move closer to Adjuvant! after the oncology visit?*

We used McNemar's test to assess the degree to which the patient estimates improved. McNemar's test compares the number who improved with the number that got worse, with respect to our ± 5% accuracy threshold. It uses the Binomial distribution to test whether the number of improved estimates is equal to the number that got worse, where the number of trials is the total of improvements and deteriorations; improvement is a success; and the probability of success is 0.5 under the null hypothesis.

For both study questions, we performed sensitivity analysis on our definition of accuracy, testing the robustness of our findings under scenarios where we focused on recurrence alone, mortality alone, and either recurrence or mortality. We also performed sensitivity analysis on our accuracy threshold to establish whether our findings for pre-visit patient beliefs were different for a margin of ± 10% than for ± 5%. Finally, we conducted two-way sensitivity analysis, simultaneously varying both the definition of accuracy and the threshold.

## Results

### Sample

Twenty out of 21 consecutive, eligible women consented after being offered participation in this study. The woman who did not participate did not state a reason for declining. Eighteen women (90%) were White and most (n = 17, 85%) were U.S.-born. Their ages ranged from 40 to 76 years old, with a median age of 54 years. A plurality of respondents were married or were living with a partner (n = 15, 75%), were college graduates (n = 9, 45%), were employed full-time (n = 10, 50%), and had an annual family income between $75,000 and $150,000 (n = 7, 35%). The two attending oncologists participating in the study (including author HSR) confirmed that all of the consenting patients fell into the subgroups for which Adjuvant!'s accuracy had been validated as being within 5% [9].

Table 1 enumerates the recurrence and mortality estimates, respectively, for the 20 patients. The Adjuvant! model forecasted a broad range of local therapy recurrence and mortality estimates, displayed by increasing recurrence risk in the second column of Table 1. This range reflected the heterogeneity of our participants with respect to age, tumor size, tumor grade, node involvement, hormone receptor status, and comorbidities.

**Table 1:** Local therapy recurrence and mortality estimates

Recurrence estimates (%)						Mortality estimates (%)				
ID	A!	P (B)	P (A)	P-A! (B)	P-A! (A)	A!	P (B)	P (A)	P-A! (B)	P-A! (A)

6	12	5	50	-7	38	39	5	50	-34	11
11	13	90	5	77	-8	4	10	0	6	**-4**
12	14	50	10	36	**-4**	9	30	5	21	**-4**
19	14	10	20	**-4**	6	28	10	10	-18	-18
10	15	15	20	**0**	**5**	6	10	10	**4**	**4**
3	15	10	50	**-5**	35	12	10	20	**-2**	8
20	16	30	15	14	**-1**	8	20	0	12	-8
5	17	15	25	**-2**	8	22	5	20	-17	**-2**
16	18	30	15	12	**-3**	14	30	10	16	**-4**
17	19	30	20	11	**1**	8	10	5	**2**	**-3**
2	20	95	20	75	**0**	9	90	0	81	-9
4	21	30	20	9	**-1**	15	10	20	**-5**	**5**
8	24	40	20	16	**-4**	13	10	10	**-3**	**-3**
18	26	30	25	**4**	**-1**	17	10	10	-7	-7
9	33	90	65	57	32	24	90	75	66	51
1	34	20	10	-14	-24	23	5	10	-18	-13
13	39	0	40	-39	**1**	27	0	25	-27	**-2**
7	40	30	40	-10	**0**	28	10	40	-18	12
15	53	70	70	17	17	41	70	40	29	**-1**
14	95	100	90	**5**	**-5**	89	100	80	11	-9

This table shows the difference between patient Adjuvant! estimates, before and after the oncology visit. Patient estimates highlighted in boldface are within ± 5% of Adjuvant! Abbreviations are ID for study identification number, A! for Adjuvant!, P for patient, (B) for before the visit, and (A) for after the visit.

### Analysis

1. *Did patient estimates of local therapy recurrence and mortality match Adjuvant! estimates before the oncology visit?*

Before the visit, 2 patients out of 20 were within ± 5% of their Adjuvant! estimates for both recurrence and mortality.

Sensitivity Analysis

Table 2 summarizes our analysis of sensitivity to the 5% threshold for patient estimates before the oncology visit. Changing the ± 5% margin of accuracy to ± 10% resulted in 2 more patients matching Adjuvant! on both recurrence and mortality estimates before the oncology visit.

**Table 2:** Sensitivity analysis of threshold for margin of error and outcome measure

Recurrence and mortality									
Threshold (±)	Right before	Wrong before	Stayed right	Stayed wrong	Got right	Got wrong	Right after	Wrong after	McNemar p-value
**5%**	**2**	**18**	**1**	**12**	**6**	**1**	**7**	**13**	**0.125**
10%	4	16	3	6	10	1	13	7	0.012
**Recurrence**									
**5%**	**6**	**14**	**3**	**5**	**9**	**3**	**12**	**8**	**0.146**
10%	9	11	7	3	8	2	15	5	0.109
**Mortality**									
**5%**	**5**	**15**	**4**	**9**	**6**	**1**	**10**	**10**	**0.125**
10%	7	13	7	5	8	0	15	5	0.008
**Recurrence or Mortality**									
**5%**	**9**	**11**	**7**	**3**	**8**	**2**	**15**	**5**	**0.110**
10%	12	8	11	2	6	1	17	3	0.125

This table shows how results change moving from a ± 5% to a ± 10% accuracy threshold within an outcome measure, and how results change depending on which outcome measure is the focus of the analysis.

We also examined accuracy of patient recurrence and mortality estimates separately. Before their visit, for recurrence, 6 patients out of 20 were accurate within ± 5% of their Adjuvant! estimate. For mortality, 5 patients out of 20 were accurate within ± 5% of their Adjuvant! estimate before their oncology visit. Finally, we analyzed patient performance in estimating either recurrence or mortality. At the ± 5% accuracy threshold, 9 patients were accurate before on either one or the other or both.

2. *Did patient estimates of local therapy recurrence and mortality move closer to Adjuvant! after the oncology visit?*

Seven patients ended up within the ± 5% accuracy threshold on both recurrence and mortality estimates. Of the two who started matched within ± 5% of Adjuvant! before the oncology visit, one of them became mismatched, but 6 of the 18 who started mismatched moved into agreement with Adjuvant!, for a net total of 7 accurate patient estimates after the visit (p = 0.125, McNemar's test, based on the 6 of 7 who improved rather than worsened in accuracy).

Sensitivity Analysis

At a threshold of ± 10%, 13 matched Adjuvant! after the oncology visit for both recurrence and mortality estimates. At this threshold, the McNemar test for improvement was significant (p = 0.012, based on 10 of 11 who improved rather than worsened).

Regarding estimates of recurrence alone, 12 patients ended up within the ± 5% threshold after the visit. Of the 6 who started matched, 3 became mismatched, while 9 of the 14 who started mismatched, became matched (p = 0.146 by McNemar's test). Regarding estimates of mortality alone, 10 patients ended up within the ± 5% accuracy threshold for mortality estimates. Of the 5 who started matched before, 1 became mismatched, while 6 of the 15 who started mismatched, became matched (p = 0.125 by McNemar's test.)

We also analyzed patient performance in estimating either recurrence or mortality after the visit. At the ± 5% threshold, 9 patients were accurate before while 15 were accurate after, but the improvement was not significant (p = 0.110 by McNemar's test).

## Discussion

### Study findings

Our purpose was to generate preliminary results regarding patient beliefs about 10-year local therapy recurrence and mortality risks, before and after the use of Adjuvant! printouts during oncology consultations. Based on this study, we conclude:

1. A majority of breast cancer patients were inaccurate (i.e. beyond ± 5%) in their estimates of local therapy recurrence and mortality before seeing an oncologist who used Adjuvant! printouts;

2. The oncology visit including Adjuvant! printouts did not significantly improve the proportion who were accurate after compared to before the visit;

3. A majority of patients were inaccurate (beyond ± 5%) in their estimates of local therapy recurrence and mortality after visiting an oncologist who used Adjuvant! printouts.

### Interpretations

Our first and third findings suggest that oncologists may not be able to rely on their current practices, including printing Adjuvant! screenshots, to educate a majority of their patients to an appropriate degree of accuracy about their recurrence and mortality risks.

Our second finding is likely an artifact of our small sample size. Our results did suggest improvement in the number of patients with accurate estimates for one, either, or both recurrence and mortality outcomes.

Our findings also depend on three critical assumptions. First, we assume that Adjuvant! is indeed a gold standard for 10-year local therapy outcomes against which patient estimates can be evaluated. All three of our findings are sensitive to this assumption.

A second assumption is that patient estimates need to be within ± 5% of their surgery recurrence and mortality risks in order to be considered accurate. If we relax the threshold for accuracy from ± 5% to ± 10%, our study does detect a statistically significant improvement in the number of patients with accurate estimates where no such statistical improvement exists with the more stringent accuracy threshold. Therefore our second finding is sensitive to this assumption.

Third, we assume that patients need to understand both recurrence and mortality risks associated with local therapy, because recurrence affects quality while mortality affects quantity of life. Our insistence on including both outcomes increases expectations for risk communication compared to focusing on either recurrence or mortality alone. For example, in our study, only seven patients accurately estimated both recurrence and mortality risks after seeing their oncologist. However, 12 were accurate on recurrence afterwards and 10 were accurate on mortality afterwards. Therefore our third finding, that a majority of patients remain inaccurate, depends on whether patients need to know both recurrence and mortality, or one, or either.

### Study quality

This is the first detailed report of patient estimates about local therapy prognosis in the context of oncologists using Adjuvant! printouts as a teaching tool during the oncology visit. We have previously reported methodological insights from this study [12,13] and we have written an editorial about some of the implications [14]. Other studies have reported on the use of Adjuvant! but not its influence on patient knowledge [10,11]. A recent study simulated the hypothetical use of the Adjuvant! interface in an internet survey of women, but these women were not being treated for breast cancer [15] Another has reported on the impact of another decision aid (the Decision Board) on patient knowledge, but the Decision Board focuses on 5-year outcomes for recurrence only [16]. Our findings provide some preliminary, quantitative evidence that patients were not adequately informed about their local therapy prognoses before meeting with oncologists; and that the use of a validated and widely disseminated oncologist reference tool was not sufficient to adequately educate a majority of patients about their local therapy prognoses.

However, this study was subject to biases and its results should only be interpreted with an orientation to preliminary findings and hypothesis generation. This was a pre/post single-sample design, with no control group, so we cannot be sure that any changes in accuracy of patient estimates were due to use of the Adjuvant! printouts compared to other aspects of the oncology visit. The sample was small, so the study was underpowered to rule out the role of chance in producing apparent changes before and after the oncology visits. We asked patients only about their beliefs, so we cannot rule out the possibility that they could accurately recall risk estimates but did not believe them. We used a categorical response format that limited the resolution of patient estimates to 5% increments. This study was conducted before the availability of biologic therapies and genetic risk assessment tests. Our report does not focus on other aspects of the oncology visit, including the discussions of adjuvant therapy recurrence, mortality, and side effect rates.

### Connections to the literature

This being the first report examining patient knowledge before and after exposure to Adjuvant!, there are no directly comparable studies of Adjuvant! against which to triangulate our findings and interpretations. However, in a study of risk communication using an alternative to Adjuvant!, Whelan and colleagues tested a Decision Board that presented 5-year recurrence risk estimates (derived from registry data) to node-negative breast cancer patients. Thirty-one out of 92 patients in the control arm came within ± 10% of the gold standard estimates for recurrence risk without chemotherapy [16]. Therefore, under usual care conditions, most breast cancer patients did not match gold standard 5-year recurrence risk estimates, even with a ± 10% accuracy threshold. We also found that a majority of patients (9 out of 20) unexposed to a decision aid (in our study, at baseline) did not match 10-year recurrence risk estimates within ± 10%.

In the Decision Board intervention group, 49 out of 82 (60%) matched the gold standard within ± 10%, which was a significant improvement compared to the control group (31 out of 92 or 34%, p < 0.001). We also found an improvement in recurrence estimates, from 9/20 before to 15/20 after, at the ± 10%, although this was not statistically significant in our study. The authors did not report sensitivity analysis to their accuracy threshold, and so we cannot compare findings at our more stringent, and we believe more clinically appropriate, threshold of ± 5%.

Our findings are also consistent with the literature on risk communication outside of adjuvant therapy for breast cancer. Generally, people have difficulty retaining and recalling probabilities [4]. The Adjuvant! interface was designed before the emergence of best practices regarding the use of graphical formats in risk communication [17,18]. A recent internet survey randomized women at risk for breast cancer to either the Adjuvant! interface, versus interfaces that incorporated best practices in risk communication, and found significant improvements in retention and recall of likelihoods [15]. Our findings can also be linked to theoretical constructs. Risk estimates about local therapy prognosis are available from specialized sources such as Adjuvant!, and systematic reviews such as the Early Breast Cancer Trialists' Collaborative Group [19]. However, surgeons and others may not be communicating these estimates to patients before they arrive at their oncology visit, reflecting an implementation gap. This would partially explain why only 2 patients were well-informed about local therapy prognosis before their oncology visit.

Those patients (18 in our sample) who are inadequately informed before their oncology visit may face significant emotional and cognitive barriers to absorbing information presented during the visits [20,21]. Furthermore, even if they could overcome these barriers, they may not be the kind of information seekers who *want *to absorb prognostic information [22,23].

Those patients who do seek prognostic information may experience overload as they are presented with recurrence, mortality and side effect rates associated with hormone therapy, chemotherapy, and combined hormone and chemotherapy treatment options, as well as the local therapy baseline. For example, patients in our sample were better able to estimate one or the other risk estimate after their oncology visit than both together. Also, the Adjuvant! interface was initially designed for use by oncologists, and its bar charts and other formats may not be comprehensible or memorable enough for patients.

Finally, patients may not believe the information they are being presented. The discrepancy between their estimates and those provided by Adjuvant! may arise from the fact that patients may feel more optimistic or more pessimistic about their chances, and may adjust their estimates accordingly.

## Conclusion

Our study found that whether we hold patients to an accuracy threshold of ± 5% or ± 10% on recurrence, mortality, or both outcomes, changes our evaluation of Adjuvant! printouts. Oncologists and researchers therefore need to agree on clinically relevant thresholds for accuracy in risk communication, and on whether patients need to understand both recurrence and mortality risks (along with side effects).

Pending further studies and progress, oncologists should be aware that relying exclusively on Adjuvant! printouts to communicate both recurrence and mortality estimates to patients may leave a majority of patients misinformed. Oncologists may wish to focus initially on either recurrence or mortality, check with patients for understanding, and then assess with patients whether additional information will result in overload. Finally, oncologists should initiate or participate in research to replicate or repudiate our findings, and experiment with alternative approaches. For example, the developers of Adjuvant! could experiment with revising the software's interface to reflect the latest findings in graphical display of risk information [15].

Oncologists face the daunting task of communicating recurrence and mortality risks, for local therapy, chemotherapy, hormone therapy, and combined chemo-hormonal therapy, to patients who may be in cognitive and emotional overload. While Adjuvant! is a well-validated prognostic tool, its interface was initially intended to be used by oncologists using it as a reference, not as an educational intervention. Our study suggests a need for continued improvement in the communication of breast cancer therapy recurrence and survival risks.

## Competing interests

The authors declare that they have no competing interests.

## Authors' contributions

JKB, LJE, and HSR conceived of the study design. LJE, HSR, and DFC contributed to data acquisition. JKB, DHM, and DWH analyzed the data. All the authors were involved in drafting and critically revising the manuscript, and reading and approving the final submission.

## Pre-publication history

The pre-publication history for this paper can be accessed here:

http://www.biomedcentral.com/1471-2407/9/127/prepub
